# In vitro and in vivo evaluation of antioxidant activity, acute toxicity, and antihyperglycemic effect of individual and binary mixtures of crude methanolic extracts of *Cucurbita moschata* and *Bidens pilosa*

**DOI:** 10.1371/journal.pone.0352400

**Published:** 2026-07-17

**Authors:** Hellen Phiri, Agape Lumai, Kadango Zombe, Evelyn Funjika, Peter Mubanga Cheuka, James Nyirenda

**Affiliations:** Department of Pure and Applied Chemistry, School of Natural and Applied Sciences, University of Zambia, Lusaka, Zambia; Mustafa Kemal University: Hatay Mustafa Kemal Universitesi, TÜRKIYE

## Abstract

**Background:**

*Bidens pilosa* (BP) and *Cucurbita moschata* (CM) are used to treat diabetes traditionally. Little is known scientifically about the toxicity and efficacy of the individual or binary mixtures on blood-sugar-lowering effects. Hence, this study carried out toxicity studies and examined the antidiabetic effects of these plants to support traditional knowledge. We report the phytochemical composition, total phenolic (TPC), flavonoid content (TFC), and antioxidant activity of individual and mixed methanolic BP *and* CM leaf extracts. We also report acute toxicity of the individual extracts. This study is the first to investigate the antidiabetic effects of a combined BP and CM leaf extract in alloxan-induced diabetic mice.

**Aim:**

The study aimed to determine toxicity and the blood-glucose-lowering effects of individual and combined mixtures of BP and CM on alloxan-induced diabetic mice.

**Materials and Methods:**

Acute toxicity of the extracts was assessed through the LD_50_ assay in rats after oral administration. TFC was measured using a modified aluminium chloride colourimetric method, while TPC was measured using the Folin-Ciocalteau’s method. The antioxidant activity was analysed using the Ferric Reducing Antioxidant Power (FRAP) method, 2,2-diphenyl-1-picrylhydrazyl (DPPH), and hydrogen peroxide radical scavenging assay. We investigated the antihyperglycemic effect of the extracts by measuring their blood-glucose-lowering effect in alloxan-induced diabetic mice.

**Results:**

No mortality or significant behavioural changes were observed at doses up to 10,000 mg/kg, suggesting a high safety margin for the two plants under study. Oral administration of the combined leaf extract of BP and CM in the ratios of 1:1, 1:2, and 2:1 w/w % showed that the BP: CM (1:2) extract at the concentration of 500 mg/kg significantly reduced blood glucose levels from 17.2 ± 0.15 mmol/L to 3.9 ± 0.04 mmol/L. The positive control, glibenclamide (5 mg/kg), reduced blood glucose levels from 17.0 mmol/L to 4.1 mmol/L, respectively (p < 0.0001).

**Conclusion:**

In vivo results of this study suggest that a combined leaf extract of *B. pilosa* and *C. moschata* may provide glucose-lowering effects as seen in mice though this is yet to be tested in humans. In addition, the extract may provide a nutritional source among Zambians. Acute toxicity results suggest that *C. moschata* and *B. pilosa* extracts are essentially safe, supporting their use as food and a potential source of nutraceuticals.

## Introduction

Diabetes mellitus disrupts metabolism and leads to persistently elevated blood sugar levels. Diabetes arises from inadequate insulin production, the hormone that governs glucose levels, or the body’s diminished capacity to respond to insulin appropriately [1]. It’s a serious, chronic condition that arises when the body cannot use insulin effectively, or the pancreas cannot make enough of it [2]. There are three different types of diabetes: type I (insulin-dependent diabetes), type II (non-insulin-dependent or adult-onset diabetes), and gestational diabetes (diabetes during pregnancy). Most diabetic patients have type II diabetes.

In 1980, an estimated 108 million adults had diabetes; by 2014, that figure had risen to 422 million [3]. Since 1980, the prevalence of diabetes has almost doubled worldwide. Compared to high-income countries, the prevalence of diabetes rose more quickly in low and middleincome countries between 2006 and 2016 [2]. In 2012 alone, diabetes was the cause of 1.5 million deaths [4]. These resulted from a heightened risk of cardiovascular diseases, leg amputations, kidney failure, nerve damage, and loss of vision. All three forms of diabetes have the potential to cause complications and increase the likelihood of premature death. Heart attack, stroke, kidney failure, leg amputation, vision loss, and nerve damage are among the potential side effects. Uncontrolled hyperglycaemia during pregnancy raises the chance of foetal mortality.

Due to direct medical costs, diabetes and its complications place a heavy financial burden on people with the disease, their families, healthcare systems, and national economies [5]. According to a prevalence-based approach, the total projected cost of diabetes in the United States was $ 245 billion, which included $ 69 billion in lost productivity and $ 176 billion in direct medical expenses [6]. Healthcare providers have increasingly prescribed more expensive insulin analogues over the less expensive animal and human formulations, contributing significantly to the rise in insulin expenditures [2]. In a research conducted by Mwila, Bwembya [7], respondents admitted to having personal financial challenges because of the diabetic treatment regimen. Costs associated with purchasing the food required to comply with diabetic nutrition therapy, purchasing glucose monitoring equipment, and supplementing the hospital’s monthly prescription supply were some of the difficulties encountered.

Medicinal plants have served as an alternative treatment to overcome the problem of diabetes because synthetic medications and accessible insulin therapy cause physiological side effects such as fatty liver, insulin resistance, anorexia nervosa, and brain atrophy [8]. People use medicinal plants such as *Lannea edulis* [9,10], *Kigelia africana* [11], *Zanthoxylum chalybeum* [12], and *Cassia abbreviata* [13] as complementary treatments for diabetes mellitus. Multiple epidemiological studies have identified a link between the intake of foods high in polyphenols and a lower risk of age-related diseases in humans [14]. Researchers attribute this connection to the antioxidant properties of flavonoids and various other polyphenols. Antioxidants help shield cells from the harmful effects of free radicals. The body contains compounds that can minimise the harm caused by free radicals or prevent their development. The body obtains antioxidants from both internal processes and external sources. Internally, it produces antioxidants through the action of enzymes such as superoxide dismutase [15]. Antioxidants obtained externally come from diets that contain minerals, flavonoids, vitamins A, E, and C, as well as primarily plant-based polyphenols [16–19]. Numerous studies show that oxidative stress significantly influences the pathophysiology of chronic illnesses like diabetes mellitus [20]. It can weaken the body’s antioxidant defence system, increasing oxidative burden. Oxidative stress produces reactive oxygen species (ROS), which dysregulate key cellular functions and jeopardise cellular balance and health [21]. Because ROS elimination largely relies on internal and external antioxidant defences, individuals with chronic or degenerative diseases are more vulnerable to oxidative stress or related damage due to elevated oxidant levels and/or diminished antioxidant defences [22]. Research indicates that people with low levels of antioxidants may face a higher risk of experiencing diabetic complications such as retinopathy, nephropathy, amputations in the lower limbs, coronary artery disease, and cardiovascular conditions [23], which are the leading causes of morbidity and mortality worldwide [24].

*B. pilosa* (BP), commonly known as blackjack, is a herbaceous plant species in the Asteraceae family [25]. Experts classify it as an invasive weed that affects annual and perennial crops and spreads extensively in tropical and subtropical regions. In some nations, such as Nigeria, Benin, and Zimbabwe, the plant is grown on a small scale for food and medicinal purposes [26]. There are reports of local communities in Africa, Asia, and America using *B. pilosa* herbal preparations to treat various health conditions, including diabetes. [27]. Researchers have reported different properties of this medicinal plant in the literature. *B. pilosa* was reported to have antihyperglycemic, antihypertensive, antiulcerogenic, hepatoprotective, immunomodulatory and anti-inflammatory, antileukemic, antimalarial, antibacterial, antimicrobial, anticancer, and antioxidative [28–30].

Scientists classify *Cucurbita moschata* in the family *Cucurbitaceae* and believe it originated in America. The genus *Cucurbita* comprises approximately 20–27 species, most of which are herbaceous plants, with only five species being commonly cultivated [31]. The cultivated species are *Cucurbita argyrosperma*, *Cucurbita moschata*, *Cucurbita maxima*, *Cucurbita pepo*, and *Cucurbita ficifolia* [32]. All species in the genus *Cucurbita* produce fruits commonly called pumpkins, summer or winter squashes, or marrows, depending on the variety and region. [33]. *Cucurbita moschata* is the most heat-tolerant species within the *Cucurbita* genus and is also the most widely cultivated in tropical Africa. [34]. *C. moschata* is known to possess anti-obesity, antidiabetic, anticancer, antibacterial, antioxidative, antihypertensive, and anti-inflammatory [19,35–37].

In this paper, we report the acute toxicity of *B. pilosa* and *C. moschata* extracts and the extraction and evaluation of antioxidant activity [19] of the mixtures of *B. pilosa* and *C. moschata*. We also report the *in vivo* experiments on the effect of the extract mixtures on fasting blood sugar levels using alloxan-induced diabetic Wistar mice as models.

## Materials and methods

### Plant material and extraction

**Fig 1:**
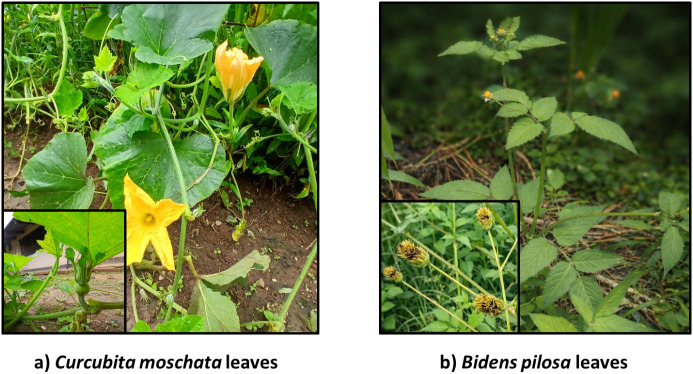
Shows the plant specimens that were deposited in the University of Zambia (UZ) Herbarium. a) *Cucurbita moschata* leaves and characteristic yellow whorl showing an inlay of the pumpkin fruit developing and in b) *Bidens pilosa* leaves showing an inlay of the characteristic pricky seeds. The two plants were positively identified by a taxonomy expert at the University of Zambia, School of Natural and Applied Sciences, Department of Biosciences and Biotechnology. Both photos were taken by the student and taxonomist at the farm where plant samples were collected.

*Bidens pilosa* and *Cucurbita moschata* plants (Fig 1) were collected at Nkungumalemba Farm (15°25’52.1” S 28°11’40.0) in Lusaka West, Lusaka Province, Zambia, in July 2022. The two plants were identified and authenticated by a qualified botanist from the University of Zambia, School of Natural and Applied Sciences, Department of Biosciences and Biotechnology, and deposited in the Institutional Herbarium with voucher specimen numbers 22485 and 22486 for *B. pilosa* and *C. moschata*, respectively.

The leaves of *C. moschata* and *B. pilosa* were washed first with tap water to remove all the dust and debris, and rinsed twice with enough distilled water as described by [38]. The clean leaves were then air-dried in the shade for 2 weeks. Dried samples were then ground to a homogeneous powder using a blender. 100 grams of powdered plant material were placed in a 2 L beaker, and 1000 mL of methanol was added. The mixture was placed on a magnetic stirrer and stirred continuously for 24 hours to ensure complete extraction. The following day, the mixture was filtered, and the filtrate was kept in a container. The remaining residue was re-extracted with another 1000 mL of methanol and filtered. The two filtrates were combined and concentrated on a Rota vapour (R-11 BUCHI, Labortechnik AG, Switzerland) at 40 °C until a semisolid mass was obtained. The extract was then placed in the desiccator for 3 days, and once completely dry, it was transferred into amber sample store bottles and kept in the refrigerator at 4 °C.

### Chemicals

The standards Gallic acid (Purity ≥ 98%), 2,4,6-tripyridyl-s-triazine (TPTZ, Purity ≥ 98%), and 2,2-Diphenyl-1-picrylhydrazyl (DPPH, Purity ≥ 95%), Folin-Ciocalteau’s reagent (Purity 98%), Alloxan monohydrate (Purity ≥ 98%), and Quercetin (Purity≥95%) were purchased from Sigma Aldrich through Merck & Co.

### Phytochemical screening

The presence of phytochemical compounds in the samples was investigated using several different methods, as described by [9,19,39,40]. The phytochemical compounds analysed included alkaloids, saponins, steroids, terpenoids, glycosides, flavonoids, phenols, tannins, carbohydrates, amino acids, and proteins.

### Quantification of phenolic compounds and flavonoids

Procedures for the quantification of total phenolic and flavonoid content are fully described in our previous paper [19,38,41]. The quantity of flavonoids in the plant extract was determined using a modified Aluminium chloride (*AlCl*_*3*_) method. Quercetin was used as the standard. Working standards with concentrations ranging from 5 µg/mL to 500 µg/mL were prepared by serial dilutions. 2 mL of either the standard or extract was mixed with 2 mL of a 2.0% *AlCl*_*3*_ solution and incubated at room temperature for 60 minutes.

To quantify phenolic compounds in the plant extracts, the Folin-Ciocalteau method was used, with some modifications. Gallic acid was used as a standard. Working standards with concentrations ranging from 5 µg/mL to 500 µg/mL were prepared by serial dilutions. 0.5 mL of each standard solution was mixed with 0.5 mL of Folin-Ciocalteau’s reagent and allowed to stand for 5 minutes. The mixture was then diluted with 3 mL of distilled water and 2 mL of sodium carbonate solution (*Na*_*2*_*CO*_*3*_). The resulting mixture was incubated at room temperature in the dark for 1 hour and 30 minutes.

The absorbances of the standards and plant extracts for both TFC and TPC were measured against the blank at 420 nm and 765 nm, respectively, with a UV-visible spectrophotometer (model UV-2600, Shimadzu).

### Antioxidant activity

Procedures for the quantification of total phenolic and flavonoid content are fully described in our previous paper [19]. Briefly, The antioxidant power of plant extracts was determined by a modified Ferric Reduction Antioxidant Power (FRAP), developed by Benzie and Strain in 1996. Ascorbic acid served as a positive control. The FRAP reagent was prepared using 0.3 mol/L (300 mmol/L) acetate buffer (pH 3.6), 10 mmol/L 2,4,6-Tris (2-pyridyl)-s-triazine (TPTZ) solution in 40 mmol/L hydrochloric acid (HCl), and 20 mmol/L irn (III) chloride prepared solution (*FeCl*_*3*_ *6H*_*2*_*O* in the ratio 10:1:1 (v/v).

The radical scavenging activity was determined using 2,2-diphenyl-1-picrylhydrazyl (DPPH) and hydrogen peroxide assay. Ascorbic acid was used as a positive control in both assays. Different concentrations of ascorbic acid and samples ranging from (0–100 µg/mL) were prepared.

The absorbances of the positive controls and plant extracts for FRAP, DPPH, and hydrogen peroxide assays were measured against the blank at 593 nm, 514 nm, and 230 nm, respectively, with a UV-visible spectrophotometer (model UV-2600, Shimadzu).

### Acute toxicity

Acute toxicity was performed on Wistar albino rats of both sexes to determine the safe dose. Extracts of *B. pilosa* and *C. moschata* were suspended in 0.9% (w/v) normal saline using ultrasonic sonication for 4 minutes. Eight groups of five rats each were created from the forty rats, and each group received oral treatment once at various doses (normal control, 10, 100, 300, 500, 1000, 5000, 10,000 mg/kg). Animals were weighed before dose administration. Animals were observed during the first 4 hours after administration and then after 24 hours for any changes in behaviour or physical activities.

The Karber method was used to determine the median lethal dose (LD_50_) [42], and for signs of toxicity, the Organisation for Economic Cooperation and Development (OECD 2000) guide was followed to determine toxicity, with the help of the Hodge and Sterner scale shown in Table 1 [43].

**Table 1:** The Hodge and Sterner toxicity scale.

Term	LD_50_ (Rat, Oral)
Extremely Toxic	Less than 1 mg/kg
Highly Toxic	1-50 mg/kg
Moderately Toxic	50-500 mg/kg
Slightly Toxic	500−5,000 mg/kg
Practically non-toxic	5,000–15,000 mg/kg
Relatively Harmless	15,000 mg/kg or more

The LD_50_ was calculated using the Karber method given in the equation below:


$$\begin{array}{c} {\text{LD}}_{\text{50}}\;=\;{\text{LD}}_{\text{100}}\;-\frac{\sum (a\;\times \;b)}{\text{n}} \end{array}$$

Where;

n = total number of animals in a group.

a = the difference between two successive doses of administered extract/substance.

b = the average number of dead animals in two successive doses.

LD_100_ = Lethal dose causing the 100% death of all test animals.

### Sugar lowering effects of plant extracts

Fifty (50) selected diabetic and non-diabetic mice were divided into ten groups (n = 5). As a normal control, Group 1 received 1 mL of 0.9% w/v normal saline orally through a feeding tube. Group 2 served as a negative control – mice had alloxan-induced diabetes and received 1 mL of 0.9% w/v normal saline orally every morning. Group 3 was used as a positive control; mice in this group developed diabetes brought on by alloxan and were given glibenclamide (5 mg/kg/day) as a reference medication every morning. Groups 4–10 served as treatment groups and had alloxan-induced diabetes. Groups 4 and 5 were given 250 and 500 mg/kg of *B. pilosa* doses, and groups 6 and 7 received 250 mg/kg and 500 mg/kg of *C. moschata* doses, respectively. The BP: CM mixture was administered in 1:1, 2:1, and 1:2 ratios at 500 mg/kg to groups 8, 9, and 10, respectively. The animals were given the extracts once every morning by oral gavage before meals. Fasting blood glucose (FBG) levels were recorded using an on-call extra glucometer (model No. OGM-191, ACON Laboratories, San Diego, USA) 72 hours after diabetes induction. The treatment was administered for fourteen (14) days. To ensure the reliability of results, quality control was performed on the on-call extra glucometer according to the instrument instructions before each test analysis.

### Animal care

In accordance with the Institutional Animal Care and Use Committee’s recommendations, all animals were anaesthetised with an acceptable dosage of 60 mg/kg of pentobarbital sodium after all experiments, and they were then sacrificed by cervical decapitation [44,45]. A qualified institutional animal attendant handled all animals, fed them, changed cages, and drew blood. After completing each of the experiments, animals were kept in the animal house for an additional 8 hours before commencing euthanasia to reduce suffering. For both toxicity and antidiabetic experiments, no animal died during the induction and experimentation period. No mortality was recorded, and for euthanasia, sleepiness, sensitivity to light, corner sitting, and drowsiness were used as humane endpoints to inform euthanasia. The carcasses after euthanasia and excess chow were incinerated at the institutional medical incinerator by the School of Veterinary Medicine, University of Zambia. Ethical clearance was obtained from the University of Zambia Natural and Applied Sciences Research Ethics Committee (REF No. NASREC: 2024-DEC-008).

## Data processing and analysis

The documented data were analysed statistically using GraphPad Prism software version 10.4.0. All the values were expressed as mean ± Standard Error of the Mean (SEM). The results were analysed for statistical significance using one-way ANOVA with Post-hoc Turkey HSD and Dunnett’s Multiple Comparisons Tests. P < 0.05 was considered significant.

## Results

### Phytochemical screening

Table 2 presents the various phytochemicals identified in the individual methanolic extracts, as well as in the simple mixtures of the two plants in the ratios 1:1, 2:1, and 1:2 (w/w). The test for terpenoids was negative for *C. moschata*.

**Table 2:** Phytochemical profile of the methanolic extracts of *B. pilosa* and *C. moschata.*

Phytochemicals	Methanolic extract				
	*B. pilosa*	*C. moschata*	BP: CM (1:1)	BP: CM (2:1)	BP: CM (1:2)
Alkaloids	+	+	++	++	++
Phenolic compounds	+	+	++	++	++
Flavonoids	+	+	++	++	++
Phyto steroids	+	+	+	+	+
Terpenoids	+	–	+	+	+
Glycosides	+	+	++	++	++
Saponins	+	+	++	++	++
Proteins	+	+	+	+	++
Amino acids	+	+	+	+	+
Reducing sugars	+	+	++	++	++
Carbohydrates	+	+	+	++	++

Phytochemical compounds present included alkaloids, phenols, flavonoids, steroids, terpenoids, glycosides, saponins, proteins, amino acids, reducing sugars, and carbohydrates. The positive test results are indicated by (+) sign, strong intensity results are indicated by (++) and negative test results are indicated by the (−) sign.

### Phenolic and flavonoid quantification assays

**Fig 2:**
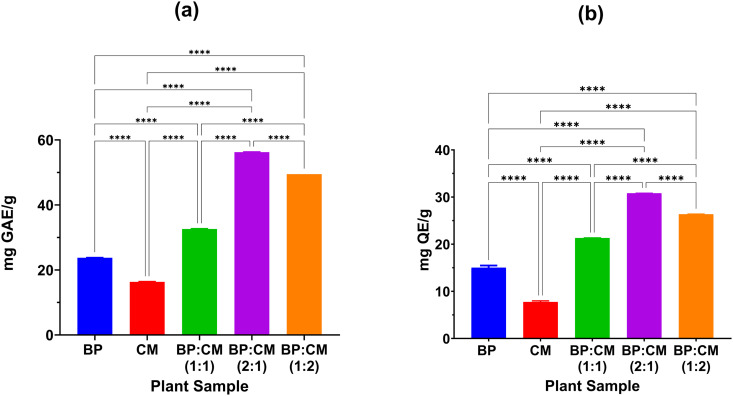
Total phenolic (a) and flavonoid (b) content of methanolic crude extracts of CM, BP, BP: CM (1:1), BP: CM (2:1), and BP: CM (1:2), respectively. The data represent the mean ± SEM of three independent experiments. Bars connected by the lines are significantly different according to Tukey’s multiple comparisons test analysis. p-values less than 0.0001 are indicated by ****.

The relative proportions of the two plants’ phenolic and flavonoid contents are displayed in Fig 2.

The results obtained from this study demonstrate that crude methanolic extracts of *Bidens pilosa* and *Cucurbita moschata*, both individually and in combination, contained appreciable levels of phenolic and flavonoid compounds.The higher phenolic content observed in *B. pilosa* may explain its relatively stronger bioactivity compared to *C. moschata*, supporting previous reports that link polyphenols with glucose-lowering potential.

### Antioxidant activity

Table 3 Shows the antioxidant activity results measured by the FRAP, DPPH, and H_2_O_2_ radical scavenging assays. The in vitro antioxidant assays revealed a concentration-dependent radical scavenging activity for all extracts, with the combined extract showing enhanced effects relative to individual extracts.This suggests a possible additive or combined effect between phytochemicals present in both plants. However, the antioxidant activity of all extracts was weawker than that of the positive control in FRAP and DPPH assays, but the B.pilosa: C.moschata in the ratio 1:2, recorder stronger antioxidant activity using hydrogen peroxide assay.

**Table 3:** Antioxidant activity of the leaf extracts of B.pilosa and C.moschata.

	FRAP	DPPH	H_2_O_2_
Sample	mM Fe (II) equiv/g	IC_50_ (µg/mL)	IC_50_ (µg/mL)
*C. moschata*	25.862 ± 0.155	65.898 ± 0.393_C_	50.255 ± 0.014_C_
*B. pilosa*	35.762 ± 0.337	35.777 ± 0.141_B_	102.208 ± 0.023_B_
*B. pilosa:C. moschata* (1:1)	46.667 ± 0.205	26.859 ± 0.212_D_	42.693 ± 0.020_D_
*B. pilosa:C. moschata* (2:1)	64.609 ± 0.077	20.401 ± 0.125_E_	38.305 ± 0.011_E_
*B. pilosa:C. moschata* (1:2)	57.804 ± 0.077	23.100 ± 0.116_F_	30.081 ± 0.005_F_

Values are expressed as Means ± SEM. Means marked by different letters are considered significant for p < 0.0001 compared to the ascorbic acid positive control for the DPPH and H_2_O_2_ assays.

Based on the measurements of FRAP, DPPH, and H_2_O_2_, the mixtures of BP and CM (1:1, 2:1, and 1:2 ratios) demonstrated a higher antioxidant activity than the individual crude BP and CM.

### Acute toxicity studies

Acute toxicity was assessed for 24 hours, and the results presented in Table 4 provide indicative doses (mg/kg).

**Table 4:** Toxicity studies on *C. moschata* and *B. pilosa* leaf extracts.

		*C. moschata*			*B. pilosa*		
Group	Dose (mg/kg)	Weight (g)	Mortality (x/N)	Symptoms	Weight (g)	Mortality (x/N)	Symptoms
Control	Saline	175	0/5	Nil	174.8	0/5	Nil
Group 1	10	175	0/5	Nil	175.6	0/5	Nil
Group 2	100	175	0/5	Nil	175.2	0/5	Nil
Group 3	300	174.8	0/5	Nil	175.4	0/5	Nil
Group 4	500	175.6	0/5	Nil	174.6	0/5	Nil
Group 5	1,000	175	0/5	Nil	174.8	0/5	Sleepy
Group 6	5,000	174.6	0/5	Nil	174.8	0/5	Sleepy
Group 7	10,000	175.2	0/5	Sleepy, Sensitive to light, Corner sitting, Drowsy	175.4	0/5	Sleepy, Sensitive to light, Corner sitting, Drowsy

From the animal study, acute toxicity was carried out with the help of the calculation of *LD*_50_. No mortality or observable adverse effects were recorded at the highest tested dose, indicating that the extracts are relatively safe in the short term. However, the absence of biochemical and histopathological assessments limits definitive conclusions regarding long-term safety and organ-specific toxicity.

### Antidiabetic activity studies

**Fig 3:**
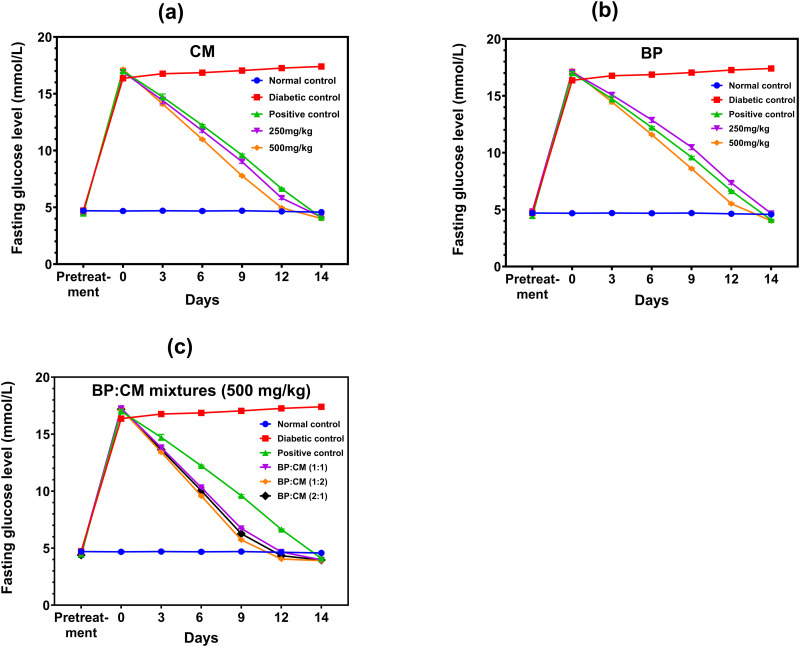
Antidiabetic effects of methanolic extracts of (a) *C. moschata*, (b) *B. pilosa*, (c) mixtures of *B. pilosa* and *C. moschata* in alloxan-induced diabetic mice for 14 days of treatment. The positive control was glibenclamide (GLB) at a 5 mg/kg body weight dose. The mixtures, 1:1, 1:2, and 2:1, were administered at a concentration of 500 mg/kg. Data is expressed as means ± SEM.

Fig 3 Shows the results collected at pre-treatment and the period covering 0 to 14 days post-alloxan induction.

The results in alloxan-induced diabetic mice demonstrated a significant reduction in blood glucose levels following treatment with the extracts. The glucose-lowering effect was dose-dependent, and although the extracts required higher doses to achieve effects comparable to the standard drug, the findings are biologically meaningful given that crude plant extracts were used.The combined extract showed improved efficacy over individual extracts, suggesting potential combined effects.

## Discussion

Phytochemical screening showed the presence of alkaloids, phenolic compounds, flavonoids, phytosteroids, terpenoids, glycosides, saponins, proteins, amino acids, reducing sugars, and carbohydrates in *B. pilosa,* while *C. moschata*, as reported by [19], was negative for terpenoids (Table 2).

Phenolic compounds are recognised for their potent antioxidant properties, stemming from their capacity to scavenge free radicals [46]. In this study, the TPC for *C. moschata* was found to be 16.342 ± 0.011 mg GAE/g. These results agree with those obtained by Al-Qaisy and Rathi (2019), who reported a TPC of 15.2 ± 0.6 mg GAE/g and 16.7 ± 0.9 mg GAE/g when the leaves were dried in sunlight and an oven, respectively. The highest TPC in *C. moschata* was recorded by Sabarudin, Zubaydah [47], who recorded TPCs of 401.4 mg GAE/g, 564.3 mg GAE/g, 368.6 mg GAE/g, and 320 mg GAE/g methanolic, ethyl acetate, *n*-hexane, and water fractions, respectively. Kim, Hong [48] also recorded a higher TPC value, 29.62 ± 0.88 mg GAE/g, than the one obtained in this study. Lusiana, Prasetyaning [49] and Kwak and Ju [50] recorded lower polyphenol content, 0.093 mg GAE/g and 2.634 ± 0.075 mg GAE/g, respectively, in *C. moschata* than this study’s findings. The TPC in the methanolic extract of *B. pilosa* was found to be 23.742 ± 0.011 mg GAE/g. Ruiz-Reyes, Mendoza-Cevallos [51] recorded higher TPC values when researchers used different extraction methods, 39.009 ± 0.450 mg GAE/g and 48.609 ± 0.370 mg GAE/g using Soxhlet extraction at 40 °C and 50 °C respectively; 30.271 ± 0.503 mg GAE/g and 40.368 ± 0.156 mg GAE/g using ultrasonic bath at 40 °C and 50 °C respectively; and 27.035 ± 0.562 mg GAE/g and 30.307 ± 0.512 mg GAE/g using maceration at 40 °C and 50 °C respectively. Higher TPC values of *B. pilosa* leaves were also recorded by Nguyen, Vu [25] and Abdulkader, Sharaf [52], who recorded TPC values of 55.97 ± 0.48 mg GAE/g and 338 ± 0.76 mg GAE/g, respectively. Lower TPCs than those obtained in this study were recorded by Kviecinski, Felipe [53] and Areekul and Phomkaivon [54], who recorded TPC values of 2.19 ± 0.2 mg GAE/g and 11.39 ± 0.35 mg GAE/g, respectively. According to the findings, phenolic compounds are crucial parts of the methanolic extracts of *B. pilosa* and *C. moschata*, and their presence may be responsible for some of their pharmacological actions. The results also revealed that the polyphenolic content of the combination of *B. pilosa* and *C. moschata* methanolic extracts in the ratios 1:1, 2:1, and 1:2, 32.621 ± 0.022, 56.250 ± 0.008, and 49.488 ± 0.008 mg GAE/g, respectively, was significantly (p < 0.05) higher than that of *C. moschata* and *B. pilosa* extracts.

The TFC of the methanolic extract of *C. moschata* was found to be 7.751 ± 0.133 mg QE/g of the dry sample weight. The TFC of this study is lower than those obtained by Jayasundara, Deraniyagala [55], who obtained TFC values of 25.0 ± 1.0 mg QE/g, 40.2 ± 0.6 mg QE/g, and 21.2 ± 0.5 mg QE/g in methanolic, ethyl acetate, and acetone extracts, respectively*.* Sabarudin, Zubaydah [47] also recorded higher TFC values than the ones recorded in this study – 491 mg QE/g, 550.7 mg QE/g, 399.8 mg QE/g, and 157.7 mg QE/g in methanol, ethyl acetate, *n*-hexane, and water fractions, respectively. The TFC of *B. pilosa* in this study was found to be 15.029 ± 0.256 mg QE/g. Ruiz-Reyes, Mendoza-Cevallos [51] analysed the TFC of *B. pilosa* leaves using different extraction methods and recorded TFC values of 17.795 ± 0.0644 mg QE/g and 16.849 ± 0.0322 mg QE/g using Soxhlet extractions at 40 °C and 50 °C respectively; 17.665 ± 0.0042 mg QE/g and 16.585 ± 0.0218 mg QE/g using ultrasonic bath at 40 °C and 50 °C respectively; and 13.986 ± 0.0218 mg QE/g and 12.554 ± 0.2573 mg QE/g using maceration at 40 °C and 50 °C respectively. Cortés-Rojas, Chagas-Paula [56] also determined the flavonoid content of B. *pilosa* using different extraction methods. They recorded TFC values of 21.988 ± 0.127 mg QE/g, 19.623 ± 0.100 mg QE/g, 16.482 ± 0.180 mg QE/g, and 11.590 ± 0.042 mg QE/g using maceration, Soxhlet, ultrasound, and microwave extraction methods. A mixture of *B. pilosa* and *C. moschata* in the ratios 1:1, 2:1, and 1:2 possessed higher flavonoid content, 21.321 ± 0.014, 30.804 ± 0.008, and 26.370 ± 0.004 mg QE/g, than the individual plants. A key bioactive characteristic of flavonoids is their ability to act as antioxidants. It can be concluded that the mixture is a good source of natural antioxidants. As a result, it can provide protective benefits against chronic illnesses while functioning as a collective rather than separately [57].

Antioxidant activity (Table 3) was carried out using the FRAP, DPPH, and hydrogen peroxide assays and the BP: CM (2:1) mixture showed the significantly (p < 0.05) highest reducing power (64.609 ± 0.077 mmol Fe (II)/g), followed by the 1:2 (57.804 ± 0.077 mmol Fe (II)/g), 1:1 (46.667 ± 0.205 mmol Fe (II)/g) mixtures. *B. pilosa* and *C. moschata* had much lower reducing powers – 35.762 ± 0.337 mmol Fe [58]/g and 25.862 ± 0.155 mmol Fe [58]/g, respectively. These results align with the findings from the DPPH assay, indicating that the antioxidant mechanisms of *B. pilosa* and *C. moschata* differ, as they are capable of donating both protons (as shown by the DPPH assay) and electrons (as indicated by the FRAP assay). Ascorbic acid was a positive control and recorded a FRAP value of 225.244 ± 0.268 mmol Fe [58]/g. Reducing power was lower for all three species when compared with ascorbic acid. Angelini, Matei [59] observed that the leaf methanolic extract of *B. pilosa* recorded the highest reducing power, 0.732 ± 0.065 mmol Fe [58]/g, compared to the root and stem extracts. However, the FRAP value Angelini et al. (2021) recorded is lower than that recorded in this study (Table 3, left panel). Adedapo, Jimoh [60] also recorded lower FRAP values than this study, 2.432 ± 0.144 mmol Fe [58]/g, 0.562 ± 0.011 mmol Fe [58]/g, and 0.036 ± 0.0001 mmol Fe [58]/g in the acetone, methanol, and water extracts of *B. pilosa,* respectively. Kim, Hong [48] concluded that the leaf extract of *C. moschata* had the highest ferric-reducing antioxidant power, 388 ± 31 mmol Fe [58]/g, than any other part, and this value is higher than what was recorded in this study.

The DPPH (Table 3, third column) scavenging activity increased with concentration for both standards and extracts. Ascorbic acid served as a positive control. Percentage scavenging activity at 100 µg/mL concentrations of standard ascorbic acid and methanolic extracts of *C. moschata, B. pilosa*, BP: CM (1:1), BP: CM (2:1), and BP: CM (1:2) were found to be 91%, 62%, 77%, 85%, 89% and 87% respectively. One of the factors that can be utilised to assess the antioxidant activity of a compound is the half maximal inhibitory concentration (IC_50_) value, which refers to the concentration of the sample capable of decreasing free radicals by 50%. The IC_50_ value is inversely proportional to the free radical scavenging activity. The lower the IC_50_ value, the more potent the substance at scavenging DPPH, and this implies a higher antioxidant activity. Dunnett’s Multiple Comparisons Tests were used in a one-way ANOVA to assess the data’s statistical significance. Means denoted by a different letter were considered significant for p < 0.0001 when compared to the positive control.

The results were compared to standard ascorbic acid, which served as a positive control, with an IC_50_ value of 16.319 ± 0.109 µg/mL. The results of this study revealed that *B. pilosa*: *C. moschata* mixtures are more potent than individual *B. pilosa* and *C. moschata* extracts, and it can be deduced that BP: CM mixtures can act as a primary antioxidant based on their capability to scavenge DPPH free radicals. The IC_50_ value of *C. moschata* extract was found to be 65.898 ± 0.393 µg/mL, which was categorised as strong antioxidant activity. Research by Pujiastuti, Erwiyani [61] concluded that the ethanol extract of pumpkin fruit has potent antioxidant activity with an IC_50_ value of 69.00 µg/mL. Research conducted by Sabarudin, Zubaydah [47] recorded lower IC_50_ values, thus more powerful antioxidant activity, in *C. moschata* leaves than those obtained in this study: 8.832 ± 1.429 µg/mL, 6.737 ± 0.196 µg/mL, 9.679 ± 0.543 µg/mL, and 11.805 ± 0.63 µg/mL in the methanol, ethyl acetate, *n*-hexane, and water fractions, respectively. On the other hand, Kim, Hong [48] and Suresh [62] recorded weaker antioxidant activity in *C. moschata* extracts, IC_50_ of 1270 ± 140 µg/mL and 435 µg/mL, respectively, than this study reported.

*B. pilosa* also showed strong antioxidant activity with an IC_50_ value of 35.777 ± 0.141 µg/mL against DPPH. Singh, Passsari [63] recorded weaker antioxidant activity in *B. pilosa* leaf extract, IC_50_ = 80.45 μg/mL, than in this study. Adedapo, Jimoh [60] reported a DPPH IC_50_ value of 94,200 µg/mL, which was higher than our reported value. Deba, Xuan [64] reported that the antioxidant activity of essential oils from *B. pilosa* showed that leaves and flowers had DPPH IC_50_ values of 47 and 50 μg/mL, respectively, further demonstrating that, in comparison to other plant parts, leaves exhibited the strongest antioxidant potential. Cortés-Rojas, Chagas-Paula [56] obtained IC_50_ values consistent with those of this study: 36.408 ± 2.352 µg/mL using ultrasound extraction and 36.072 ± 0.429 µg/mL using the microwave extraction method. Stronger antioxidant activity in *B. pilosa* extract was also recorded by Cortés-Rojas, Chagas-Paula [56], 17.805 ± 0.704 µg/mL using maceration extraction, and 27.012 ± 1.192 µg/mL using the Soxhlet extraction method.

The results of the hydrogen peroxide radical scavenging assay of methanolic crude extracts of the plant species are in Table 3, fourth column. The scavenging activities of the 100 μg/mL extracts, from highest to lowest, were as follows: BP: CM (1:2) mixture (82%), BP: CM (2:1) mixture (74%), BP: CM (1:1) mixture (69%), *C. moschata* (65%), and *B. pilosa* (44%). Ascorbic acid was used as the positive control and recorded an IC_50_ value of 37.197 ± 0.006 µg/mL. The BP: CM mixture extracts showed significant (p < 0.0001) radical scavenging activity against hydrogen peroxide when compared to ascorbic acid. Both plant species showed hydrogen peroxide scavenging activity when compared to ascorbic acid. The naturally present H_2_O_2_ in the atmosphere, water, and human body exists in low concentration levels. It rapidly breaks down into oxygen (O_2_) and water (H_2_O) and may generate hydroxyl radicals (OH), which can trigger lipid peroxidation and lead to DNA damage [65]. Extracts of plant species used in this study effectively scavenged hydrogen peroxide, likely due to the presence of phenolic groups that can donate electrons to hydrogen peroxide, thus neutralising it into water (H_2_O).

*B. Pilosa* and *C. moschata* leaves have been part of a regular dish in Zambia since time immemorial, but toxicity information has been lacking or unreported using scientifically sound methodologies. To ensure the safety of the two plants, the extracts underwent a toxicity test based on the Hodge and Sterner toxicity scale (Table 1), with doses up to 10,000 mg/kg; practically no animals died, even at this extremely high dose. The LD_50_ values for these two plants were therefore regarded as exceeding 10,000 mg/kg (Table 4), and following the Hodge and Sterner toxicity scale detailed in Table 1, these extracts are classified as being in the practically nontoxic range.

An experiment was carried out to deliver estimated amounts of individual extracts or simple ratio combinations of the extracts in order to evaluate the effectiveness of managing diabetes mellitus as reported by locals [10,19]. The effect of repeated oral administration of crude extracts on blood glucose levels in alloxan-induced diabetic mice is shown in Fig 3. Prior to the onset of diabetes, blood glucose levels in each treatment group were normal and did not differ statistically from one another (p > 0.05). To ascertain whether the variation in blood glucose levels was statistically significant with p < 0.05, a one-way analysis of variance [66] was employed. Mean blood glucose levels after induction of diabetes (day 0 blood glucose levels) of the positive control, diabetic control, and treatment groups rose to above 17 mmol/L, and they were significantly different (p < 0.0001) when compared to the normal control group. The results revealed that fasting blood sugar was reduced more potently in mice receiving a mixture of *B. pilosa* and *C. moschata* (1:2) (500 mg/kg), which significantly (p < 0.0001) reduced the glucose concentration in comparison to the diabetic control group. The test extract activity was dose-dependent for the individual plant extracts (Fig 3a and b), with *C. moschata* showing a higher potency than *B. pilosa* for the two concentrations studied. The glucose level in the diabetic control group steadily increased from the beginning to the end of the experiment. Initially, the blood glucose level of the untreated diabetic control group was 16.36 ± 0.16 mmol/L, and after 14 days, the blood glucose level increased to 17.4 ± 0.09 mmol/L. In comparison to the normal control group, the diabetes control group experienced a significant increase in mean blood glucose levels (p < 0.0001) on all survey days following alloxan induction. The mean fasting glucose levels of the positive and extract treatment groups on days 3 and 14 were significantly different (p < 0.0001) from day 0 when compared to the diabetic control, as shown in Fig 3.

The results revealed that the plant extracts may have antidiabetic properties. A wide range of phytochemical compounds derived from various plant species are believed to possess notable hypoglycaemic and glucose-lowering properties. In a study conducted by Kifle, Yesuf [67],It was shown that bioactive compounds such as flavonoids and phenols exert their glucose-lowering effects through multiple mechanisms, including stimulating insulin secretion from pancreatic β-cells, reducing glucose absorption in the small intestine, enhancing glucose uptake and utilisation in body tissues, and promoting glycogenesis in the liver. Flavonoids have also been demonstrated to up-regulate two peroxisome proliferator-activated receptors, resulting in glycemic and lipid regulation required for the management of diabetes [29]. Hsu, Lee [68], found that giving streptozotocin-induced diabetic mice a daily dose of *B. pilosa* water extract, either once or over a 28-day period, decreased blood glucose levels and raised serum insulin levels. Bartolome, Villaseñor [69] demonstrated that the mechanism of action of *Bidens pilosa* includes influencing the immune system in rats, as it safeguards the β-cells from antibodies produced by the body.

On the other hand, Chaturvedi, Singla [70] demonstrated that both the water extract and the protein-bound polysaccharide extract of *C. moschata*, when administered to alloxan-induced diabetic rats and mice, resulted in improved glucose tolerance and increased insulin levels while decreasing fasting blood glucose. Chaturvedi, Singla [70] linked these outcomes to a decline in hepatic beta-oxidation and a reduction in lipid production within the body. Therefore, the ability of the plant extracts in this research to lower glucose levels may be linked to the flavonoids and phenolic compounds found in the extracts. Glibenclamide is commonly utilised as a reference antidiabetic medication in alloxan-induced diabetes for evaluating the effectiveness of plant extracts. In this study, results indicated that *B. pilosa* (500 mg/kg) and *C. moschata* (250 mg/kg) showed a significant antihyperglycemic effect at higher doses, which is almost the same as that of glibenclamide (Fig 3). This is in agreement with Ajagun-Ogunleye, Tirwomwe [71], who also found that the 800 mg/kg extract of *B. pilosa* had significant hypoglycemic effects that were slightly comparable to those of glibenclamide. Glibenclamide has demonstrated antidiabetic impact, likely by promoting insulin secretion from β-cells in the pancreas and suppressing glucagon release [72].

Additionally, flavonoids derived from natural sources have been demonstrated to stimulate insulin secretion from pancreatic β-cells [73]. In addition to promoting insulin secretion, flavonoids also reduce the production and spread of free radicals, which helps decrease oxidative stress and subsequently protects against complications associated with diabetes. Given that *B. pilosa* and *C. moschata* contain these secondary metabolites, the hypoglycemic effects of the extracts may be linked to a mechanism comparable to that of glibenclamide. Phytochemical screening revealed that all investigated plant extracts tested positive for alkaloids, but they were only present in high quantities in *B. pilosa* and *C. moschata* mixtures. Alkaloids are known to influence glycemic regulation by enhancing the accessibility of blood glucose to peripheral tissues and managing oxidative status, which helps prevent the onset of complications related to diabetes [29]. Alkaloids assist pancreatic β-cells in secreting more insulin, which regulates blood sugar levels and restores antioxidant status, both vital factors for preventing complications associated with diabetes [74]. Therefore, the ability of the plant extracts to lower blood sugar levels in this study may be attributed to the presence of alkaloids. The oral administration of the combined leaf extract of *B. pilosa* and *C. moschata* in the ratios of 1:1, 1:2, and 2:1 (Fig 3c) significantly reduced fasting blood glucose to normal levels from day 3 to day 9. The decrease was gradual from day 12 to day 14. When compared with individual plant leaf extracts, the combination extracts were more potent and suggest a combined or additive behaviour of glucose-lowering effects. Thus, the combined extracts had stronger antihyperglycemic and antioxidant properties than the individual extracts. The effects of the combined leaf extracts of *B. pilosa* and *C. moschata* persisted throughout the study period. However, there is a need for human clinical trials as almost all research has examined alloxan- or streptozotocin-induced models of diabetes.

## Conclusion

Despite the promising findings, this study has several limitations that should be acknowledged.

The crude methanolic extracts from *B. pilosa* and *C. moschata* were only evaluated in an acute toxicity model, which, despite showing no mortality at the highest dose administered, does not adequately confirm long-term safety; therefore, future research should include sub-chronic and chronic toxicity assessments, along with detailed evaluations of vital organ function, hematological parameters, and biochemical measures. Additionally, the antihyperglycemic activity was demonstrated using crude extracts that required relatively large doses to achieve glucose-lowering effects comparable to the standard drug, indicating the need for bioassay-guided fractionation to isolate and identify the active constituents responsible for the observed effects. The study was also restricted to an alloxan-induced diabetic mouse model, which may not fully replicate the complex pathophysiology of human diabetes, suggesting the value of evaluating the extracts in additional animal models and, eventually, clinical settings. Furthermore, while antioxidant activity was verified, the underlying mechanistic pathways related to the antihyperglycemic effects were not examined, and future studies should look into molecular targets such as insulin signaling, glucose uptake processes, and oxidative stress indicators. Finally, this work is the first to assess the combined leaf extract of *B. pilosa* and *C. moschata*, but whether the extracts possess synergistic or antagonistic interactions between the two plants remain unclear and warrant further investigation to optimize their potential as antidiabetic nutraceuticals*.*

## Supporting information

S1 File Supporting information phiri et al 2025. (XLSX)
